# An *in vitro* model of granuloma-like cell aggregates substantiates early host immune responses against *Mycobacterium massiliense* infection

**DOI:** 10.1242/bio.019315

**Published:** 2016-08-03

**Authors:** Sungmo Je, Hailian Quan, Yirang Na, Sang-Nae Cho, Bum-Joon Kim, Seung Hyeok Seok

**Affiliations:** 1 Department of Microbiology and Immunology, andInstitute of Endemic Disease, Seoul National University College of Medicine, Seoul 110-799, Republic of Korea; 2 Department of Microbiology, andInstitute for Immunology and Immunological Diseases, Yonsei University College of Medicine, Seoul 120-752, Republic of Korea

**Keywords:** *Mycobacterium massiliense*, Host immune responses, *In vitro* granuloma, PBMC

## Abstract

*Mycobacterium massiliense* (*M. mass*), belonging to the *M. abscessus* complex, is a rapidly growing mycobacterium that is known to cause tuberculous-like lesions in humans. To better understand the interaction between host cells and *M. mass*, we used a recently developed *in vitro* model of early granuloma-like cell aggregates composed of human peripheral blood mononuclear cells (PBMCs). PBMCs formed granuloma-like, small and rounded cell aggregates when infected by live *M. mass*. Microscopic examination showed monocytes and macrophages surrounded by lymphocytes, which resembled cell aggregation induced by *M. tuberculosis* (*M. tb*). *M. mass*-infected PBMCs exhibited higher expression levels of HLA-DR, CD86 and CD80 on macrophages, and a significant decrease in the populations of CD4+ and CD8+ T cells. Interestingly, low doses of *M. mass* were sufficient to infect PBMCs, while active host cell death was gradually induced with highly increased bacterial loads, reflecting host destruction and dissemination of virulent rapid-growing mycobacteria (RGM). Collectively, this *in vitro* model of *M. mass* infection improves our understanding of the interplay of host immune cells with mycobacteria, and may be useful for developing therapeutics to control bacterial pathogenesis.

## Abstract (precis)

**Summary:** An *in vitro* model of granuloma-like cell aggregates infected with *Mycobacterium massiliense* improves our understanding of the interplay of host immune cells with mycobacteria.

## INTRODUCTION

*Mycobacteriaum abscessus* (*M. abscessus*) complex are rapidly growing mycobacteria (RGM) that are important human pathogens responsible for a wide spectrum of soft tissue infections and disseminated infection in immunocompromised patients (Prevots and Marras, 2015). *M. abscessus* complex are closely involved in respiratory infection, especially in patients with cystic fibrosis or chronic bronchiectasis (Olivier et al., 2003). *M. abscessus* complex comprises three genetically related species: *M. abscessus* (*sensu stricto*), *M. massiliense*, and *M. bolletii* (Zelazny et al., 2009). In a clinical study, *M. abscessus* was found to acquire inducible resistance to clarithromycin more commonly than *M. massiliense* (*M. mass*), and treatment response rates to antibiotic therapy including clarithromycin were much higher in patients with *M. mass* than in those with *M. abscessus* (Koh et al., 2011).

*M. mass* is known to exhibit either smooth or rough colony morphologies on solid media (Kim et al., 2012, 2013) depending on specific mutations of genes associated with the synthesis of glycopeptidolipid (GPL) (Kim et al., 2013; Howard et al., 2006). The rough strains produce cord-like structures involved in *in vivo* pathogenesis and immune evasion (Bernut et al., 2014). Furthermore, in mice, rough variants were shown to be hypervirulent when administrated intravenously compared to the smooth wild-type (Catherinot et al., 2007). Thus, each colony morphotype of mycobacteria exhibits different interactions between the bacteria and host immune system.

Similar to virulent mycobacteria like *M. tuberculosis* (*M. tb*) and the *M. avium* complex (MAC), *M. mass* also causes inflammatory diseases, ranging from localized abscess to disseminated lesion (Shang et al., 2011; Martins de Sousa et al., 2010). In a mouse model, *M. mass* induced granulomatous responses involving the activation of macrophages, dendritic cells, and natural killer cells in host immune cells (Martins de Sousa et al., 2010). *M. abscessus*- or *M. mass*-infected macrophages secrete inflammatory cytokines and chemokines, such as TNF-α, IL-12, IL-6 and MCP-1, which recruit and activate various immune cells (Martins de Sousa et al., 2010; Kim et al., 2014a; Shin et al., 2008). Recruitment and activation of mononuclear cells and T lymphocytes leads to early granuloma formation in infection sites, like the lung, spleen and liver.

Granuloma formation is a major defense response against mycobacterial infection to prevent dissemination. Granulomas are initially composed of activated macrophages including epithelioid cells, surrounded by T and B lymphocytes (Silva Miranda et al., 2012). This progresses into a mature granuloma with fibrosis by fibroblast recruitment, or caseous necrosis later. Recently, *in vitro* models of granuloma using PBMC were developed to study the molecular interactions between host immune cells and mycobacteria (Puissegur et al., 2004; Birkness et al., 2007). These *in vitro* granuloma-like cell aggregates can be induced by infection of human or bovine PBMCs with live mycobacteria or mycobacterial antigen (e.g. PPD-coated sepharose beads). *In vitro* mycobacterial granuloma was characterized with the progressive recruitment of macrophages and T lymphocytes around the live bacilli, and they differentiated into epithelioid cells (EC) and multinucleated giant cells (MGC) within about 7 days after infection (Puissegur et al., 2004). Although *in vitro* granulomas do not exhibit some features of granuloma maturation, like fibrosis or caseousness, this model mimics early host immune responses against *M. tb* infection very well.

In this study, we first report that infection of human PBMC with *M. mass* induces the formation of granuloma-like cellular aggregates and these aggregates feature characteristics of early interactions between host cells and RGM. By using this model, we could examine early immune responses against *M. mass*, and understand the bacterial pathogenesis of smooth or rough strains, including the survival and dissemination of virulent RGM at early stages of infection.

## RESULTS

### Formation of granuloma-like cellular aggregates by infection of human PBMC with *Mycobacterium massiliense*

**Fig. 1.**
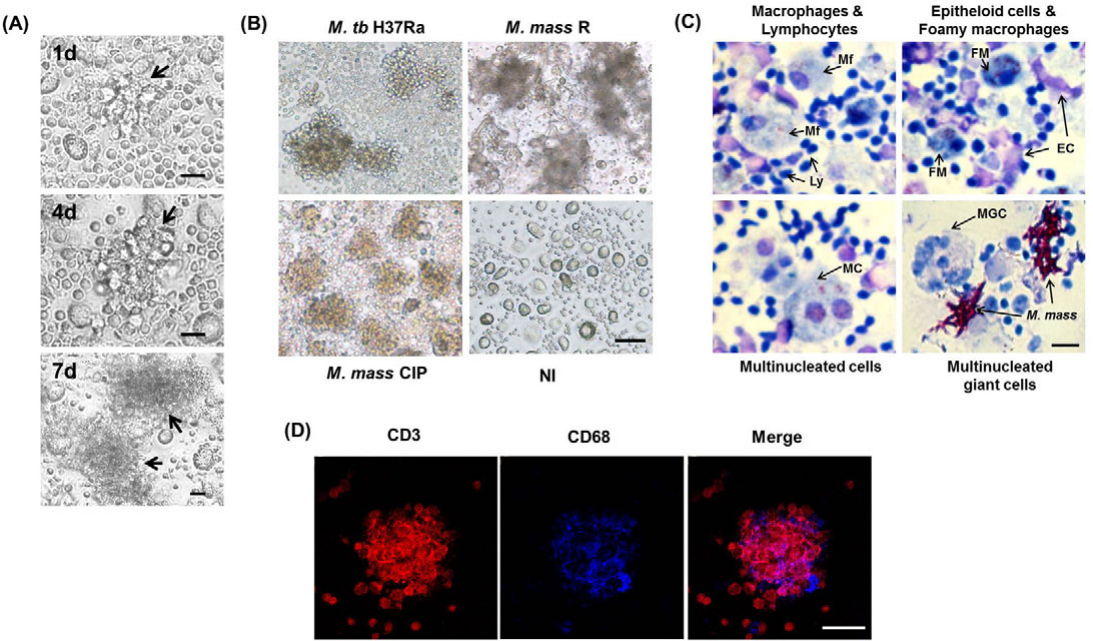
**Formation of granuloma-like cellular aggregates by infection of human PBMCs with *M. mass*.** (A) Microscopic examination of granuloma-like aggregates infected with *M. mass* at 1, 4 and 7 days after infection. Scale bar: 200 µm. Arrow indicates cellular aggregate. (B) Microscopic examination of granuloma-like aggregates infected with *M. tb* H37Ra, *M. mass* R and *M. mass* CIP at 7 days after infection. NI, non-infected. Scale bar: 400 µm. (C) Cell populations in *M. mass*-infected cellular aggregates at 7 days after infection. Giemsa and AFB staining show infected PBMCs are composed of activated macrophages (Mf), lymphocytes (Ly), epitheloid cells (EC), foamy macrophages (FM), multinucleated cells (MC) having 2 nuclei, multinucleated giant cells (MGC) having several nuclei. Scale bar: 50 µm. (D) Confocal microscopy examination of a *M. mass* CIP-induced granuloma-like aggregates stained with monoclonal antibodies against CD68 (macrophages, blue) and CD3 (T cells, red). Scale bar: 50 µm.

When fresh PBMC (1×10^6^ cells/well) were infected with mycobacteria (10^3^-10^4^ CFU/well), the cells clustered to form granuloma-like aggregates. To analyze not only structural changes but also immunological changes and differentiation of macrophage over the course of infection progression, we incubated PBMC without growth factors for 4 days and then infected them with mycobacteria. Formation of granuloma-like cellular aggregates was observed at 4 days post-infection (Fig. 1A). However, a low number of cellular aggregates with small size were examined at 4 days, and these became larger as incubation progressed. While 10^4^ colony-forming units (CFU) of *M. tb* H37Ra stably induced cellular aggregation, the same inoculum of *M. mass* induced such rapid bacterial growth that we could not keep the cellular aggregates stable for more than 7 days post-infection. Instead, 10^3^ CFU of *M. mass* was shown to form more stable aggregation, lasting more than 7 days (Fig. 1B). Microscopic examination showed that the cellular structure of the aggregates consisted of active and epitheloid macrophages surrounded by T lymphocytes. *M. mass* infection also induced the formation of foamy macrophages, a distinct characteristic of granulomas associated with virulent mycobacteria (Peyron et al., 2008). This structure has close similarities to an early form of granuloma. AFB staining of infected cells showed that the mycobacteria were phagocytosed or attached to macrophages. However, multinucleated cells (MC) and multinucleated giant cells (MGC) were sparsely observed in *M. mass*-infected PBMCs at very low frequencies (<1 of 1×10^5^), unlike virulent *M. tb*-infected granulomas (Fig. 1C) (Lay et al., 2007). Confocal microscopic examination revealed that the inner cells of the aggregates were positive for CD68, a macrophage-specific marker, and the outer cells were positive for CD3, a T cell marker (Fig. 1D). Collectively, these observations indicate that the cellular structure of the aggregates mimics early granulomas.

### Structural changes of granuloma-like cellular aggregates after *M. mass* infection

**Fig. 2.**
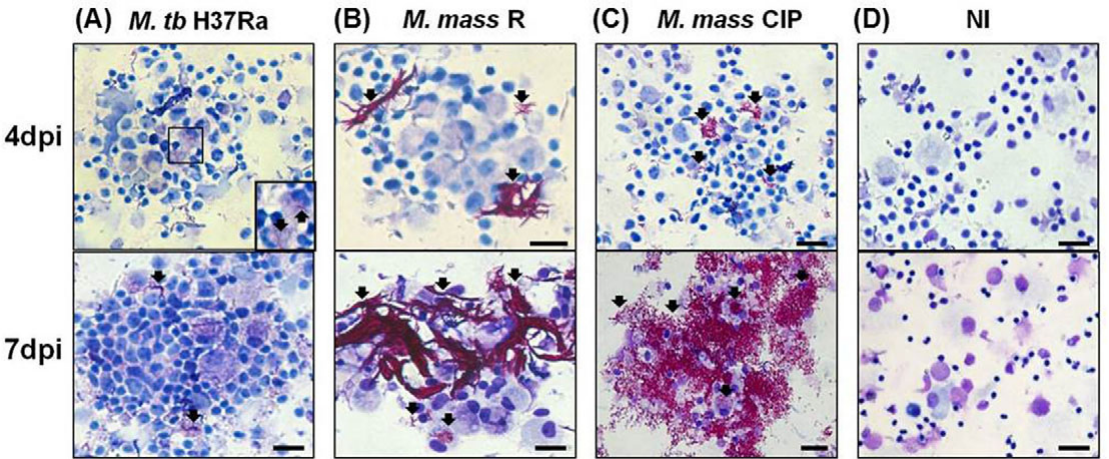
**Structural characteristics of granuloma-like aggregates after *M. mass* infection.** AFB and Giemsa staining of the cellular aggregates at 4 and 7 days post-infection (dpi) with (A) *M. tb* H37Ra, (B) *M. mass* R and (C) *M. mass* CIP. Enlarged view of mycobacteria within macrophages. (D) NI, non-infected cells (×200). Scale bar: 100 µm. Arrow indicates mycobacteria.

With continued incubation, infected cell aggregates became more common and more clearly visible. *M. tb* H37Ra infection induced larger and more compact aggregates at 7 days compared to 4 days post-infection (average size=242.8 µm, s.d.=58.4 µm) (Fig. 2A). AFB stain showed an increased number of *M. tb* H37Ra associated with macrophages at 7 days post-infection. When PBMC were infected by *M. mass*, not only intracellular but also extracellular bacterial growth was observed. To determine the main source of extracellular bacteria, we investigated the number of cell-associated or extracellular *M. mass* during infection. Cell-associated bacteria comprised most of total bacterial load and the percentage of extracellular bacteria was very low until 4 days post-infection. At 7 days, extracellular bacteria rapidly increased, accompanying cell lysis response, and showed the similar numbers to that of cell-associated bacteria (Fig. S1A,B). These results reveal that most of extracellular bacteria may come from infected cells after cell necrosis. At 7 days post-infection, extracellular rough isolate of *M. mass* (*M. mass* R) was shown to form cord-like structures, which are known to form in cultures of rough isolates of *M. abscessus* complex and other mycobacteria (Fig. 2B) (Jönsson et al., 2013a,b). The cord-like structure was found to be surrounded by host cells and induce aggregation of cells and extracellular bacteria (average size=1100 µm, s.d.=424.3 µm). *M. mass* strain CIP 108297 (*M. mass* CIP) also revealed excessive extracellular growth at 7 days, and attachment to host cell aggregates (average size=328.8 µm, s.d.=49.7 µm). Because the aggregates were surrounded by bacteria, they showed rough surface morphology under microscopy (Fig. 2C). These results indicate that virulent RGM, such as *M. abscessus* complex, may have extracellular growth or biofilm formation in susceptible hosts and interact with host cells in different ways from intracellular bacteria.

### Expression of cell surface antigens on macrophages in *M. mass*-infected PBMC

**Fig. 3.**
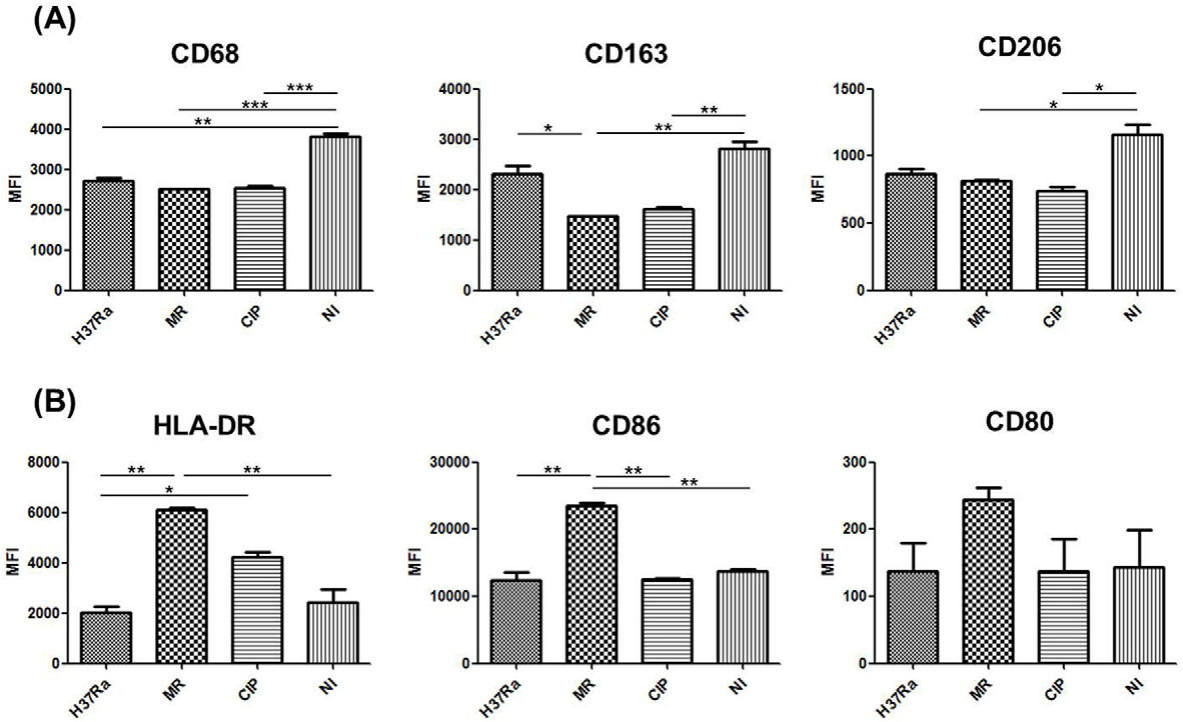
**Expression levels of cell surface antigens on macrophages in *M. mass*-infected PBMCs.** Infected and uninfected samples were harvested at 7 days post-infection and analyzed for expression of surface markers on macrophages by flow cytometry. (A) The expression levels of CD68, CD163 and CD206. (B) The expression levels of HLA-DR, CD86 and CD80. H37Ra, *M. tb* H37Ra; MR, *M. mass* R; CIP, *M. mass* CIP; NI, non-infected. One representative data set is shown out of eight unrelated healthy donors. Results are shown as the mean±s.d. from duplicated samples. *P<0.05; **P<0.01; ***P<0.001 by one-way ANOVA with Bonferroni's post-test.

To characterize the differentiation and activation of macrophages after *M. mass* infection, we investigated the expression levels of cell surface markers (CD68, CD206, CD163, HLA-DR, CD86 and CD80) on macrophages by FACS analysis. At 1 day post-infection, there was no significant difference in the expression levels of cellular markers on macrophages among the groups. At 7 days post-infection, *M. mass*-infected cells showed lower expression of CD68 (pan-macrophage marker present on lysosomal-associated membrane proteins), CD206 (mannose receptor) and CD163 (scavenger receptor) than non-infected cells (Fig. 3A). These results show that *M. mass* infection prevents the expression of anti-inflammatory markers but induces macrophage differentiation at early infection stages. The expression levels of inflammatory markers HLA-DR (MHC class II cell surface receptor), CD86 and CD80 (costimulatory signals for T cell activation) were highest on macrophages infected by *M. mass* R (Fig. 3B). In particular, the expression level of HLA-DR was significantly higher in *M. mass*-infected cells than *M. tb*-infected cells. At 4 days post-infection, the expression patterns of cellular markers on macrophages were similar to those at 7 days after infection. However, because cellular aggregates were small in number and induced a weak immune response at 4 days, we could not observe any significant difference of the expression levels between infected samples and uninfected control (data not shown). Collectively, *M. mass* infection was shown to induce surface markers related to adaptive immunity activating specific T cells and differentiate macrophages to inflammatory type rather than anti-inflammatory type at early stages of granuloma formation.

### Changes in T cell population in *M. mass*-infected PBMC

**Fig. 4.**
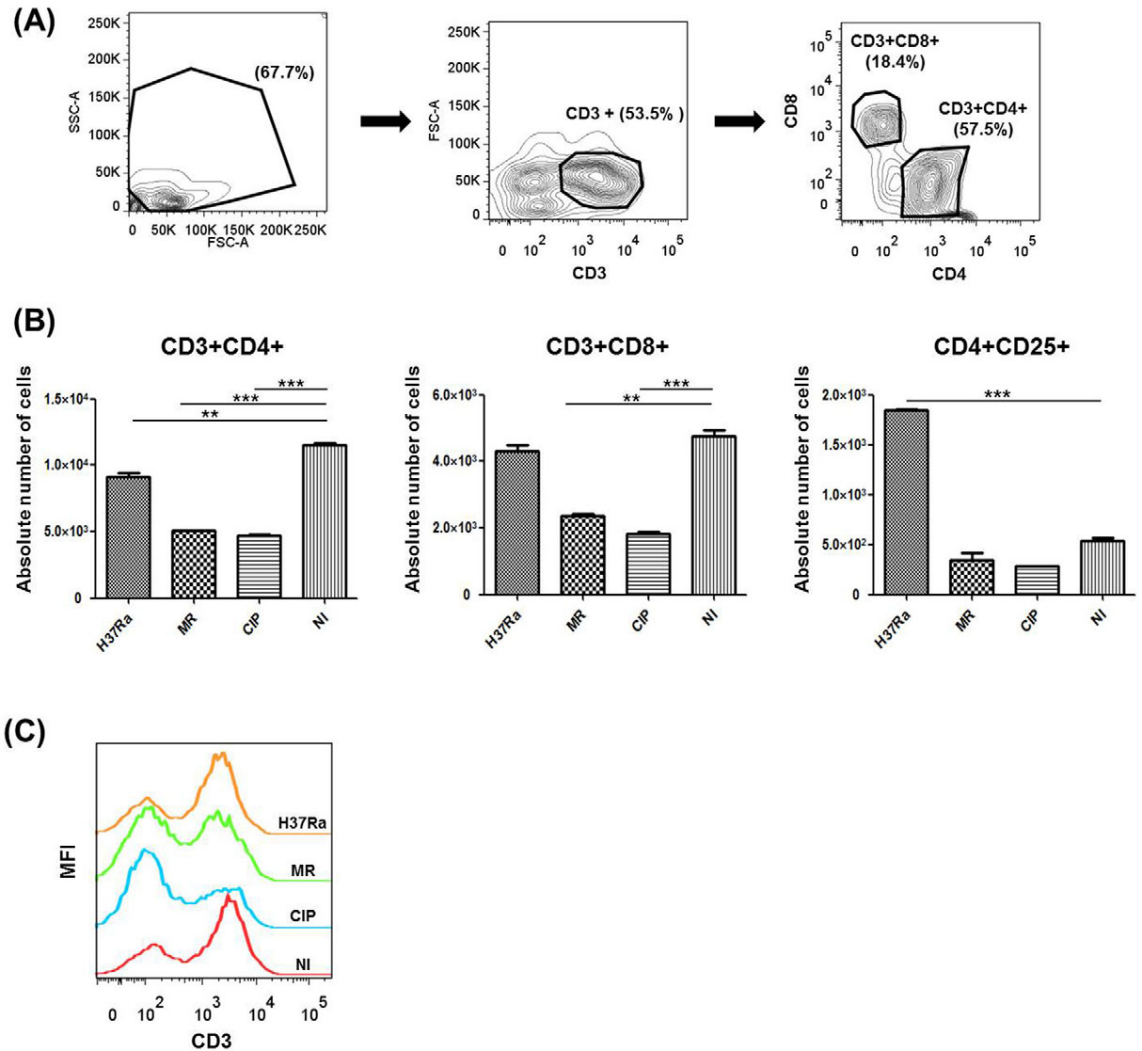
**Changes in the T cell populations of PBMC after *M. mass* infection.** At 7 days after infection, infected or uninfected PBMCs were analyzed for expression of T cell markers (CD3, CD4, CD8 and CD25) by flow cytometry. (A) Gating strategy for flow cytometry analysis of T lymphocytes. (B) CD3+CD4+, CD3+CD8+ and CD4+CD25+ T cells population in PBMCs. (C) The expression levels of T cell surface antigen CD3 measured by MFI. H37Ra, *M. tb* H37Ra; MR, *M. mass* R; CIP, *M. mass* CIP; NI, non-infected. One representative data set is shown out of eight unrelated healthy donors. Results are shown as the mean±s.d. from duplicated samples. **P<0.01; ***P<0.001 compared to non-infected group by one-way ANOVA with Bonferroni's post-test.

We investigated the population of T lymphocytes in PBMC after mycobacterial infection (Fig. 4A). *M. tb* infection is known to cause depletion of CD4+ T cells (Wu et al., 2009; Kapoor et al., 2013). Our experimental model also showed similar results. While there was no significant difference between each group at 4 days post-infection, a decrease in the population of CD3+CD4+ T cells was detected in PBMC infected by *M. tb* H37Ra at 7 days post-infection (*P*=0.048, *M. tb*-infected versus uninfected). *M. mass* infection also induced significant decrease in CD3+CD4+ T cells (Fig. 4B). While there was no change in the population of CD3+CD8+ T cells in *M. tb* infection, a significant decrease was observed in *M. mass*-infected cells with the largest decrease with *M. mass* CIP infection. *M. tb* infection is known to cause increase in CD4+CD25+ regulatory T cells (Kapoor et al., 2013). Our result also showed *M. tb* H37Ra infection increases the number of CD4+CD25+ T cells compared to uninfected control. However, *M. mass* infection did not induce a significant change in population of CD4+CD25+ T cells. In addition, we discriminated between granuloma-involved T cells and surrounding T cells, and analyzed each cell population (Fig. S2A,B). *M. mass* infection induced a decrease in CD4+ T cells and CD8+ T cells in not only granuloma-involved cells but also surrounding cells. We did not observe a significant change in CD4+CD25+ regulatory T cells by *M. mass* infection. However, *M. tb* H37Ra infection increased CD4+CD25+ regulatory T cells in granuloma-involved cells, but did not induce similar response in surrounding T cells. The decline of CD4+ T cells and CD8+ T cells by mycobacterial infection was more severe in surrounding cells. Thus, in our model, as *M. mass* infection progresses, CD3 expression levels in PBMC were reduced, accompanying increase in bacterial burden (Fig. 4C). These results indicate that *M. mass* causes a depletion of host T cells at early stages of infection to enable bacteria to effectively evade host protective responses, including adaptive immunity.

### Inflammatory cytokine secretions in *M. mass*-infected PBMC

**Fig. 5.**
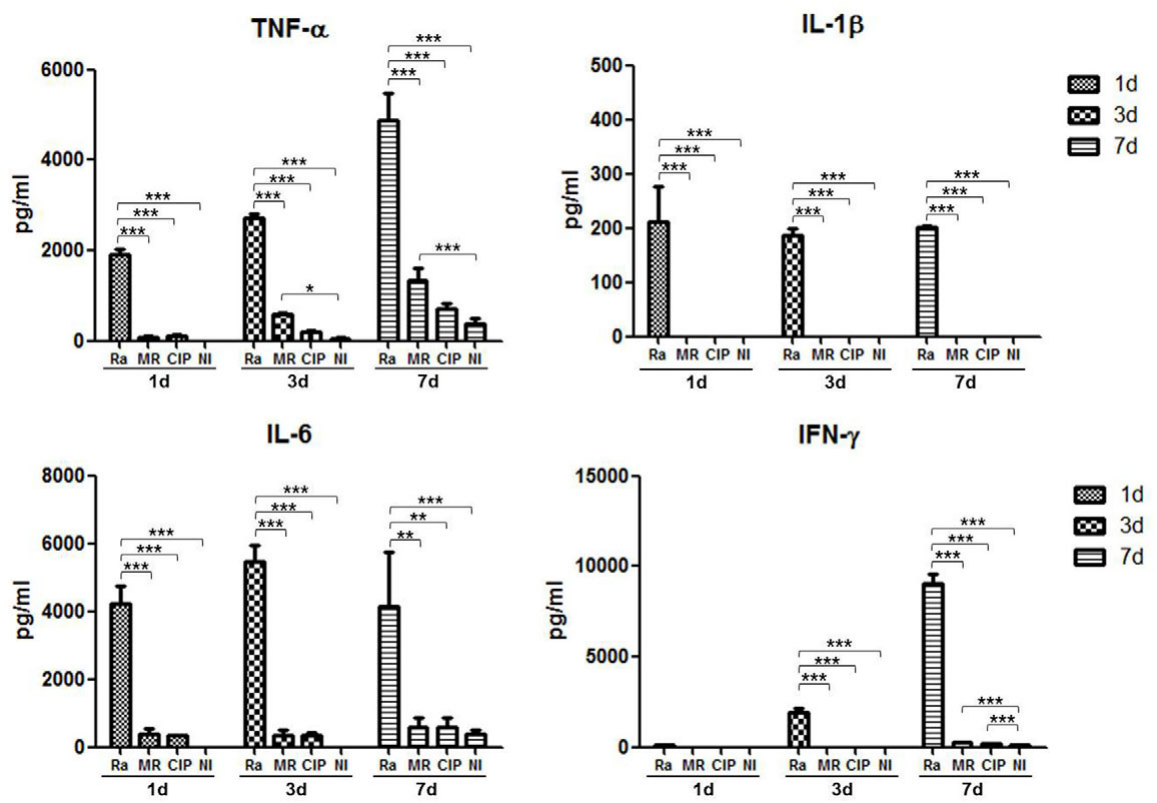
**Inflammatory cytokine secretions of human PBMC infected with *M. mass*.** Culture supernatants from different groups were harvested at 1, 3 and 7 days post-infection. Mean concentrations of secreted TNF-α, IL-1β, IL-6 and IFN-γ were measured by ELISA. Ra, *M. tb* H37Ra; MR, *M. mass* R; CIP, *M. mass* CIP; NI, non-infected. One representative data set is shown out of eight unrelated healthy donors. Results are shown as the mean±s.d. from duplicated samples. *P<0.05; **P<0.01; ***P<0.001 compared to *M. tb* H37Ra-infected group or NI group by two way ANOVA with Bonferroni's post-test.

To compare the secretion of inflammatory cytokines in response to various mycobacteria, 1×10^6^ PBMC were infected with the same inoculum of 1×10^4^ CFU/well. The culture supernatants were harvested at 1-7 days post-infection and levels of cytokines were determined using ELISA (Fig. 5). The secretion of inflammatory cytokines TNF-α, IL-1β, IL-6 and IFN-γ were induced in human PBMC from 1 day after infection. The levels of TNF-α, IL-6 and IFN-γ tended to increase gradually as infection progressed. *M. tb*-infected cells secreted significantly higher levels of inflammatory cytokines than uninfected cells at 3 or 7 days post-infection (*P*<0.001). *M. mass* led to an increase in TNF-α level compared to uninfected samples 3 days post-infection (*P*<0.05). *M. mass* R induced more TNF-α secretion than *M. mass* CIP did. IL-1β was rarely detected in PBMC infected with *M. mass* at low multiplicity of infection (MOI). IFN-γ secretion gradually increased during infection and was detected in *M. mass*-infected PBMC 7 days post-infection. Notably, although infected PBMC contained much higher bacillary loads of *M. mass* than *M. tb*, *M. mass*-infected PBMC induced lower levels of inflammatory cytokines than *M. tb*-infected cells. These results indicate that *M. mass* induces pro-inflammatory cytokines but may modulate inflammation at early stages of infection even though growing rapidly. These responses may associate with impaired maturation of *M. mass*-induced granuloma.

### Cell death in human PBMC induced by *M. mass* infection

**Fig. 6.**
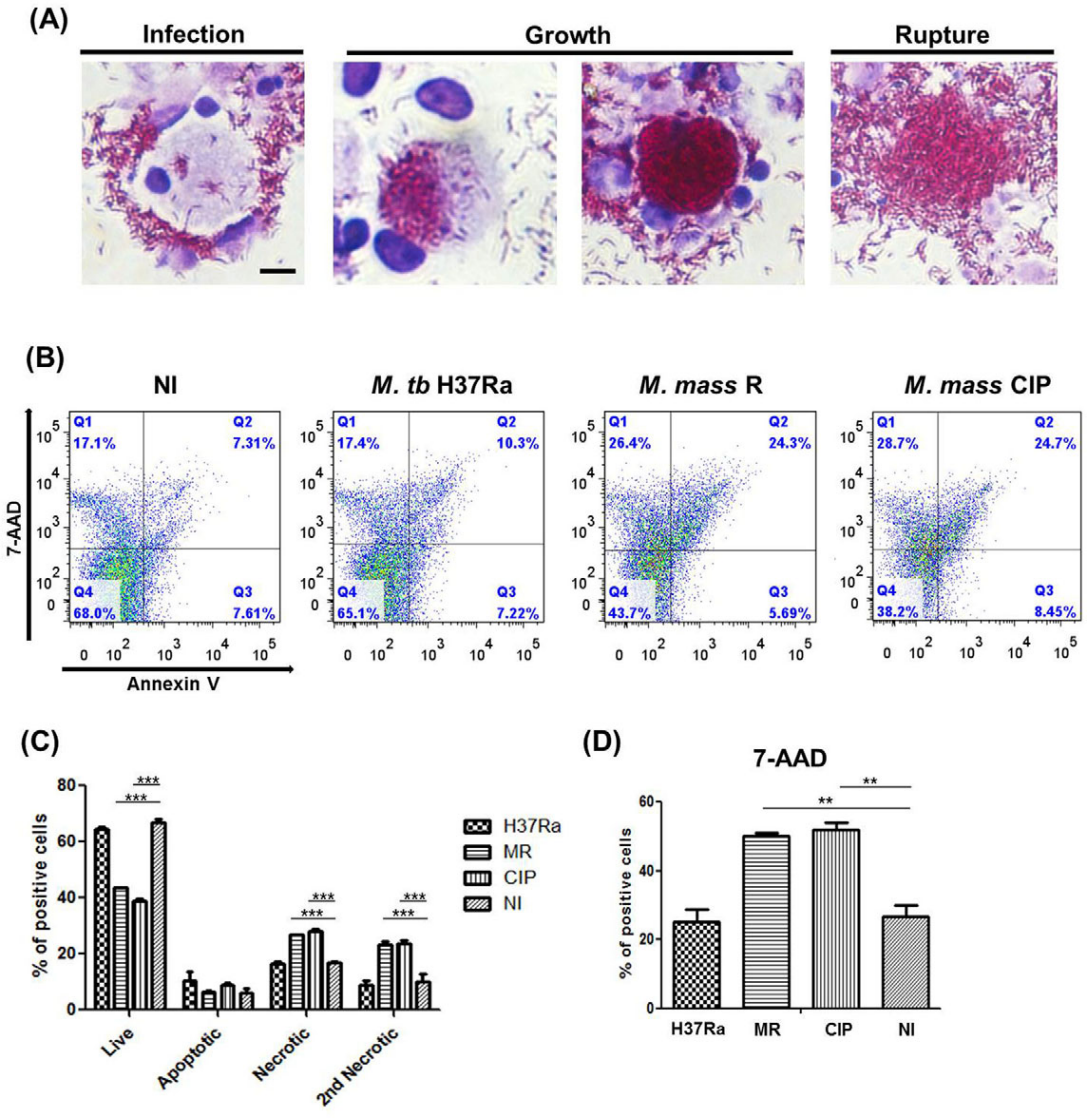
**Cell death of human PBMCs induced by *M. mass* infection.** To determinate cell death by *M. mass* infection, harvested cells from different groups were stained by Giemsa staining for microscopy and stained with Annexin V and 7-AAD for analysis by flow cytometry. (A) Microscopic examination of macrophage cell death with intracellular growth of *M. mass* CIP at 7 days post-infection, by using AFB-Giemsa staining. Scale bar: 25 µm. (B) Flow cytometry analysis of PBMC stained with Annexin V (AV) and 7-AAD at 7 days post-infection. The numbers indicate the percentage of cells in each quadrant. Lower left, AV–/7-AAD–, live cells; lower right, AV+/7-AAD–, apoptotic cells; upper left, AV–/7-AAD+, necrotic cells; upper right, AV+/7-AAD+, late apoptotic or secondary necrotic cells. (C) Bar graph representing the mean proportion of live, apoptotic, necrotic or late apoptotic cells undergoing secondary necrosis analyzed in B. (D) Bar graph representing the mean proportion of 7-AAD+ cells from each group. H37Ra, *M. tb* H37Ra; MR, *M. mass* R; CIP, *M. mass* CIP; NI, non-infected. One representative data set is shown out of eight unrelated healthy donors. Results are shown as the mean±s.d. from duplicated samples. **P<0.01; ***P<0.001 compared to non-infected group by ANOVA with Bonferroni's post-test.

Virulent mycobacteria are known to induce host cell lysis after infection. Upon microscopic examination, we observed that *M. mass* also induces death of infected cells with a high burden of intracellular bacteria (Fig. 6A). AFB staining showed that *M. mass* grows actively in infected macrophages and triggers cell membrane rupture. *M. mass* exhibited more rapid intracellular growth and stronger cytolytic activity than *M. tb* H37Ra. To determine whether *M. mass* directly induces cell necrosis or apoptosis, infected PBMCs were isolated and assessed by Annexin V and 7-AAD staining (Fig. 6B). At 4 days post-infection, we observed ruptured cells with a high burden of *M. mass* upon microscopic examination, but double staining results did not show significant differences with cell death between *M. mass*-infected and uninfected cells (data not shown). At 7 days, over 20% of *M. mass*-infected cells showed secondary necrotic features (Annexin V-positive and 7-AAD-positive) compared to about 10% of uninfected cells (Fig. 6C). In addition, the 7-AAD-positive cell population was twofold higher in *M. mass*-infected cells than *M. tb* H37Ra-infected or uninfected cells (Fig. 6D). Furthermore, the live cell population was significantly lower in *M. mass*-infected samples than uninfected samples. These results suggest that *M. mass* grows to a critical threshold and then triggers the death of host cells with more necrotic features than apoptosis. This may have detrimental effects to the host.

## DISCUSSION

*M. abscessus* complex is the most pathogenic RGM species, causing a wide spectrum of inflammatory diseases, including chronic lung disease, and skin and soft tissue abscesses. Host responses against *M. abscessus* infection mainly comprise cell-mediated immunity (CMI) (Kapoor et al., 2013). CMI responses include activation of phagocytosis, antigen presentation and antigen-specific cytotoxic T cell responses. These responses stimulate cells to secrete a variety of cytokines and chemokines (e.g. IFN-γ, IL-12, TNF-α, IL-8) that induce migration and close interaction of immune cells, leading to granuloma formation (Silva Miranda et al., 2012). Therefore, a study of mycobacteria-induced granuloma can provide us with an understanding of the molecular interactions between host immune cells and mycobacteria.

To study granulomatous response to mycobacterial infection, animal models such as mouse, guinea pig, rabbit and monkey are often used. Animal models may reproduce several of the processes occurring in humans, but some differences are often observed (Guirado and Schlesinger, 2013). For example, while mouse models are inexpensive and their genes are easy to manipulate, they do not show caseous necrosis, granuloma structure and organization. To fill this gap, several recent studies have reported models of *in vitro* granuloma formation using PBMCs treated with mycobacterial antigen-coated beads or infected with mycobacteria (Puissegur et al., 2004; Birkness et al., 2007). This model has some disadvantages inherent to *in vitro* models, but it has increased our knowledge about early cell differentiation and interactions between host cell and mycobacteria within the granuloma (Peyron et al., 2008; Guirado and Schlesinger, 2013).

In this study, we showed that *M. mass*, a species of *M. abscessus* complex, induces formation of *in vitro* granuloma-like aggregates in PBMCs. In previous studies, when PBMCs (1×10^6^ cells/well) were incubated with viable *M. tb* or *M. bovis* at MOI of 1:0.01 or higher, *in vitro* granulomas were obtained and kept stable for 14 days post-infection (Puissegur et al., 2004; Peyron et al., 2008). However, using RGM-like *M. smegmatis*, a low MOI (<1:0.01) was sufficient for inducing *in vitro* granuloma formation (Peyron et al., 2008). Because *M. mass* is also rapid-growing, we used a low MOI of 1:0.001 and could keep cellular aggregates stable for 7 days post-infection.

Granuloma-like cellular aggregates were first observed 4 days post-infection. However, cellular aggregates were small in the number and size, and immunological changes were not obvious at that point. At 7 days post-infection, we observed larger cellular aggregates, which were attached by large amounts of extracellular bacteria. When we analyzed the amounts of extracellular bacteria and cell-associated bacteria during infection, most of the extracellular bacteria appeared to have escaped from infected macrophages after cell necrosis. A previous study also reported that *M. abscessus* escapes from infected macrophages after cell death and develops extracellular growth leading to cord formation (Bernut et al., 2014). This phenomenon is different from that of *M. tb*-induced *in vitro* granulomas, which showed granuloma maturation with limited extracellular bacterial growth over infection progression (Jönsson et al., 2013a; Kapoor et al., 2013). While *M. mass* CIP was examined intracellularly and extracellularly among cell aggregates, *M. mass* R induced extracellular cord formation surrounded by host cell aggregates. Cord formation is characteristic of *M. tb* and other mycobacteria infection (Darzins and Fahr, 1956). Rough strains of *M. abscessus* complex are also known to induce cord formation, which are involved in virulence and survival of bacteria in the host environment (Bernut et al., 2014; Sánchez-Chardi et al., 2011). A study using a zebrafish model revealed rough strains of *M. abscessus* form cord-like aggregates *in vivo* and could not be phagocytosed by host cells (Bernut et al., 2014). Similarly, in our model, a high burden of extracellular bacteria and cord structure seem to have detrimental roles to host cells, and may affect reduced T cell survival. In another study, when human PBMCs were infected with a high dose of *M. abscessus*, bacterial cord formations were found to be entrapped by monocytes and lymphocytes, aggregated onto DNA extracellular traps at day 1 (Jönsson et al., 2013b). Our results also show a similar interaction between PBMCs and *M. mass* R and confirm this mechanism of mycobacterial evasion of phagocytosis, although host cells were infected with a lower dose of bacteria.

*M. mass* is known to induce granulomatous reaction and secretion of inflammatory cytokines in mice and macrophages (Martins de Sousa et al., 2010; Kim et al., 2014a). In our model, inflammatory cytokine secretion was also examined in PBMC infected with *M. mass*. However, because we infected the cells with *M. mass* at very low MOI (0.001), the levels of cytokine secretion gradually increased and seem to be lower compared to a previous study using high MOI (Jönsson et al., 2013b). Interestingly, although *M. mass* show intracellular and extracellular growth with infection progression, *M. mass*-infected PBMCs were shown to induce lower levels of inflammatory cytokines compared to cells infected with *M. tb* H37Ra. We did not use virulent *M. tb* strains, like H37Rv, and therefore did not compare the level of cytokines induced by *M. mass* to *M. tb*. However, a previous study comparing the effect of H37Ra with H37Rv infection on chemokine and cytokine expression in mouse peritoneal macrophages, H37Ra was found to preferentially induce higher levels of mRNAs of IFN-inducible protein (IP-10), lymphotactin, macrophage inflammatory protein-3 (MIP-3), and fractalkine, but there were no significant differences in genes encoding pro-inflammatory cytokines (TNF-α, IL-1β, IL-12 and IL-6) (Freeman et al., 2006). Another study using mouse peritoneal macrophages also showed no significant differences in IL-1β, IL-10, IL-12p40 and IL-12p70 secretion between *M. tb* H37Rv and H37Ra infection (Singh and Goyal, 2013). From these published results we can infer that inflammatory cytokines are induced at lower levels in *M. mass* than *M. tb* infection, suggesting that *M. mass* may have protective mechanisms modulating early inflammatory responses despite the high burden of bacteria. Granuloma disorganization can be caused by impairing pro-inflammatory cytokine production during granuloma formation (Silva Miranda et al., 2012). The modulation of early inflammatory cytokine secretion by *M. mass* may impair maturation of functional granuloma and facilitate dissemination of bacteria. Such responses may be affected by some components produced in *M. mass*. For example, GPL, a cell wall component of *M. abscessus*, was reported to block induction of human macrophage TNF-α by preventing interaction between cell wall antigen with TLR2 (Rhoades et al., 2009). To definitively elucidate the immunomodulatory strategies of *M. abscessus* complex, we need to further study the functions of specific antigens and bacterial structures, like biofilm and cord formation, in evading host immune responses.

*M. tb* infection was reported to hamper differentiation of human monocyte to macrophage, decreasing CD68 and CD36 (scavenger receptor) expression (Castaño et al., 2011). We also observed lower expression levels of CD68, CD206 and CD163 on monocytes of PBMC infected by *M. mass*. These results indicate that *M. mass* prevents monocyte differentiation into macrophages and may inhibit the activation of immune cells. HLA-DR and CD86 are known to perform major functions of antigen presentation and T cell activation (Effros et al., 1983; Jeannin et al., 2000). While HLA-DR and CD86 expression were also decreased in human monocytes infected by virulent *M. tb* H37Rv, the expression levels were increased in *M. mass*-infected cells. These results indicate that adaptive immunity associated with specific T cell activation is central to protective responses against *M. mass* (Kim et al., 2014b).

In a clinical study, *M. tb* infection was found to cause CD4+ T cell lymphocytopenia without changing levels of CD8+ T cells (Wu et al., 2009). The decrease in CD4+ T cells was also manifested in an *in vitro* granuloma model using virulent *M. tb* strains (Kapoor et al., 2013). Interestingly, in our study, avirulent *M. tb* H37Ra infection also showed a decrease in the number of CD4+ T cells. In contrast, *M. mass*-induced *in vitro* granulomas exhibited a decrease in not only CD4+, but also CD8+ T cells. This response may be caused by extracellular bacterial aggregation and cord structure, which may inhibit macrophage-T cell interaction and T cell survival, and also cause toxicity or damage to host cells. A previous study also reported that extracellular *M. abscessus*, forming cord structure, impairs host immune responses such as phagocytosis and granuloma formation, and develops active growth accompanying host cell depletion (Bernut et al., 2014). Such a depletion of effective T cells may be early pathogenic responses induced by *M. mass* infection and reflect the importance of maintaining T cell populations for host protection. A previous study using a mouse model showed that active T cell recruitment occurs during the early stages of *M. abscessus* infection and that Cd3ε^−/−^ mice were severely impaired in their ability to control bacteria growth in both the liver and the spleen (Rottman et al., 2007). Another study using IFN-γ knockout mice also showed early influx of IFN-γ and CD4+ T cells is important for clearance of *M. abscessus* infection (Ordway et al., 2008). These data align with the frequent occurrence of *M. abscessus* in patients with immunodeficiency or pre-existing lesions of the respiratory tract, like cystic fibrosis (Cullen et al., 2000).

Virulent *M. tb* is known to induce cell death of monocytes and macrophages through phagosomal rupture (Simeone et al., 2012). It thus escapes host defense mechanisms and disseminates to extracellular regions or to other cells. These responses are mediated by mycobacterial secretory systems (ESX-1) and virulent antigen, ESAT-6 (Guinn et al., 2004; Gao et al., 2004). We also observed severe cell necrosis over time in PBMCs infected by *M. mass*. While *M. tb* causes cell rupture by secreting virulent factor with membrane-lysing properties, *M. mass* may trigger membrane damage by intracellular overgrowth and aggregation of phagocytosed bacilli. Interestingly, extracellular cord formation was observed in PBMCs infected with *M. mass* R and found to affect necrotic cell death, although they are not phagocytosed. These results are similar to a previous *in vivo* study using zebrafish, that demonstrated that released *M. abscessus* Rough variant from apoptotic macrophages induces abscess following extracellular cord formation (Bernut et al., 2014). However, because *M. abscessus* complex secretory antigens and virulent factors have not yet been defined in detail, more studies using genetic or proteomic approaches are required to elucidate the mechanisms for evading immune systems and damaging host cells.

This *in vitro* granuloma model of *M. mass* infection has some limitations. *M. tb*, a virulent slow-growing mycobacterium, induces granuloma formation and goes into dormancy by adapting to the host environment (Kapoor et al., 2013). In this *M. tb*-induced *in vitro* granuloma model, dormant *M. tb* can be reactivated by adding anti-TNF-α monoclonal antibody (Kapoor et al., 2013). Although infection with *M. mass*, a virulent RGM, also induces *in vitro* granuloma-like cell aggregates with inflammatory responses, it did not enter dormancy within the granuloma. Furthermore, mycobacterial infection induces inflammatory responses followed by suppressive mediators to balance immune responses during granuloma maturation (Arkhipov et al., 2013). However, it was difficult to examine the balance between pro- and anti-inflammatory responses in the *in vitro* granulomas induced by *M. mass* infection*.* In addition, while bacterial loads of *M. mass* was shown to gradually decrease in C57BL/6 and BALB/c mice, the *in vitro* model showed an increase in intracellular and extracellular bacteria, reflecting the incomplete capability of the *in vitro* model to control the growth of *M. mass* (Shang et al., 2011; Martins de Sousa et al., 2010). In this regard, extracellular bacterial growth may induce nonspecific responses, unlike the *in vivo* circumstance. However, recent studies reported extracellular *M. abscessus* also play a role in bacterial pathogenesis in patient and animal model (Bernut et al., 2014; Qvist et al., 2015).

In summary, although *M. mass*-induced *in vitro* granulomas could not completely reproduce responses during granuloma maturation, it provides basic information about host inflammatory responses during early stages of infection. Furthermore, T cell depletion and host cell death caused by intracellular and extracellular expansion of *M. mass*, confirm that adaptive immunity is important for host protection. This is substantiated by the fact that abscesses with bacterial colonization are often observed in patients with immune disorders. Thus, this *in vitro* granuloma model provides us with insight into bacterial pathogenesis and the interplay between host and mycobacteria. Such applications will help to develop effective therapeutic strategies against mycobacterial infection.

## MATERIALS AND METHODS

### Human blood samples and cell culture

Human blood samples were collected from seven BCG-vaccinated, healthy Korean volunteers between the ages of 27 and 40 (4 males and 3 female). In Korea, BCG vaccination is compulsory for children aged 0-1 years old. Blood collection and processing was performed according to the principles established by the Seoul National University Hospital Institutional Review Board (IRB), which approved our use of blood samples (Approval Number 1402-104-560). As the procedure approved by the IRB, each volunteer signed a written consent to participate to the trial according to ethical guidelines stated in the Declaration of Helsinki. Collected PBMCs were separated from whole blood by density gradient centrifugation using standard protocol with Ficoll-Paque Plus (GE Healthcare, Piscataway Township, NJ, USA). Purified PBMCs were cultured in RPMI containing 10% heat-inactivated pooled human AB serum (Sigma-Aldrich, St. Louis, MO, USA).

### Bacterial strains and growth conditions

*M. tb* H37Ra (strain ATCC 25177) was obtained from the ATCC (American Type Culture Collection, VA, USA). *M. mass* strain CIP 108297 (*M. mass* CIP) was obtained from CIP (Collection of Institut Pasteur, Paris, France). A rough isolate of *M. mass* (*M. mass* R) was kindly provided by Dr B. J. Kim (Seoul National University, Seoul, South Korea). *M. tb* H37Ra and *M. mass* were cultured in Middlebrook 7H9 medium (BD Biosciences, Franklin Lakes, NJ, USA) supplemented with 10% OADC (BD Biosciences), 0.2% glycerol and 0.05% Tween 80 (Sigma-Aldrich). For all experiments, mycobacteria were cultured in late log phase and a portion of heavy aggregates was excluded by centrifugation. Then, collected mycobacteria were homogenized and stored at −70°C. Representative bacterial vials were thawed and the number of colony-forming units (CFU) viable on Middlebrook 7H10 agar plates (BD Biosciences) were counted. Then, mycobacteria were suspended in RPMI medium containing 10% human sera, and used for infection.

### PBMC infection for the formation of cellular aggregates

Granuloma-like cellular aggregates formation was induced as previously described (Birkness et al., 2007). Freshly isolated 1×10^6^ PBMCs were plated in 24-well culture plates (Corning, Corning, NY, USA) and differentiated for 4 days in RPMI containing 10% human serum. To induce granulomatous reaction, 1×10^3^ or 1×10^4^ CFU of mycobacteria was then added to each well, and infected PBMCs were cultured at 37°C in a 5% CO_2_ atmosphere for 10 days. To detect cytokine production of host cells, 1×10^4^ CFU was used. Formation of granuloma-like cellular aggregates was examined every day under microscopy.

### Histological staining and microscopic examinations

Granuloma-like cellular aggregates were harvested and plated onto slide glasses by using a cytospin (Thermo Fisher, Waltham, MA, USA). The cells on slide glasses were fixed for 30 min in 4% PFA solution at room temperature and, to examine lipid bodies in cells, stained for 15 min with 15% Oil Red-O (Sigma-Aldrich) in isopropanol. To examine mycobacteria within infected PBMCs, an acid-fast bacilli (AFB) stain kit (BD Biosciences) was used and staining was performed according to the manufacturer's instructions. The slides were then stained for 10 min with Giemsa staining solution (Sigma-Aldrich). For fluorescence analysis, fixed granuloma cells were blocked with 1% BSA in PBS for 1 h and then stained with phycoerythrin (PE)-conjugated anti-human CD68 monoclonal antibody and allophycocyanin (APC)-conjugated anti-human CD3 monoclonal antibody (BD Biosciences) for 2 h. Stained cells were then washed and observed under a fluorescence microscope (Olympus, Center Valley, PA, USA) and confocal microscope A1 (Nikon, Minato, Tokyo, Japan).

### Bacterial counts

To measure bacterial number of cell-associated *M. mass*, after removing culture supernatants from each well, infected cells were lysed in 0.1% Triton X-100, and 10-fold serial dilutions of lysed cells were plated on Luria-Bertani (LB) agar. After 4 days, the number of colony was counted and total bacterial numbers were calculated as log10 colony forming units (CFU). The number of extracellular bacteria was measured by recovering viable *M. mass* from culture supernatants of each sample.

### Cytokine analysis

For cytokine analysis, the supernatants of each well were harvested at 1, 3 and 7 days post-infection, and stored at −80°C until use. The levels of inflammatory cytokines (TNF-α, IFN-γ, IL-6 and IL-1β) were quantified using an enzyme-linked immunosorbent assay (ELISA) kit (Roche, Basel, Switzerland). The assays are performed according to the manufacturer's instructions.

### Flow cytometric analysis of surface antigens on macrophages

For the analysis of cell surface antigens on macrophages, PBMCs were collected at 7 days post-infection. Surface antigen staining was performed with combinations of peridinum chlorophyll (PerCP)-Cy5.5-conjugated mAb against CD14 (61D3), eFluor 450-conjugated mAb against CD11b (M1/70), PE-conjugated mAb against CD163 (eBioGHl/61), PerCP-eFluor 710-conjugated mAb against CD80 (2D10.4), PE-conjugated mAb against CD86 (IT2.2), APC-conjugated mAb against HLA-DR (LN3) (eBioscience, San Diego, CA, USA), APC-conjugated mAb against CD206 (19.2) (BD Biosciences), and fluorescein isothiocyanate (FITC)-conjugated mAb against CD68 (KP1) (Santa Cruz, Dallas, TX, USA). The expression level of CD68 was analyzed on monocyte-associated surface antigens CD14+ and CD11b+ cells, and the expression levels of CD206, CD163, HLA-DR, CD86 and CD80 were analyzed on macrophage-associated surface antigens CD68+ and CD11b+ cells. The expression levels are presented as median fluorescence intensity (MFI). The fluorescence levels on the stained cells were measured using BD LSR Fortessa and the data were analyzed using FlowJo software (BD Biosciences).

### Analysis of T cell population

For staining surface markers of T lymphocytes, the combination of PE-conjugated mAb against CD3 (HIT 3a), FITC-conjugated mAb against CD4 (OKT4) (eBioscience), APC-conjugated mAb against CD8 (RPA-T8), and PE-Cy™5 Mouse Anti-Human CD25 (M-A251) (BD Biosciences), were used. To analyze the population of granuloma-involved T cells, after removing supernatant from each well, weakly adherent cellular aggregates were detached from the well and stained with the antibodies. Surrounding T cells, not involved in granuloma formation, were collected from supernatant of each sample and stained. The samples were analyzed using BD LSR Fortessa and data analysis was done using FlowJo software (BD Biosciences).

### Annexin V and 7-amino-actinomycin D (7-AAD) staining for flow cytometry

To determine cell viability and death by apoptosis or necrosis, collected PBMCs were washed and then stained with Annexin V FITC (BD Biosciences) and 7-AAD (eBioscience) for 15 min. For maximum sensitivity, cells were analyzed 30 min after the staining by using BD LSR Fortessa (BD Biosciences).

### Statistics

Statistical analysis was performed using the Student's *t*-test or ANOVA in GraphPad Prism 5 software (GraphPad, La Jolla, CA, USA). The results were shown as the means with standard deviation (s.d.). Significant difference was defined by a value of *P*<0.05.
